# The effect of the intratumoral microbiome on tumor occurrence, progression, prognosis and treatment

**DOI:** 10.3389/fimmu.2022.1051987

**Published:** 2022-11-18

**Authors:** Feng Gao, Bo Yu, Benchen Rao, Ying Sun, Jia Yu, Daming Wang, Guangying Cui, Zhigang Ren

**Affiliations:** ^1^ Department of Infectious Diseases, The First Affiliated Hospital of Zhengzhou University, Zhengzhou, China; ^2^ Jinan Microecological Biomedicine Shandong Laboratory, Jinan, China; ^3^ Gene Hospital of Henan Province, Precision Medicine Center, The First Affiliated Hospital of Zhengzhou University, Zhengzhou, China; ^4^ Henan Key Laboratory of Ion-beam Bioengineering, School of Agricultural Sciences, Zhengzhou University, Zhengzhou, China

**Keywords:** intratumoral microbiome, tumor, inflammation, immune, genetic engineering, treatment

## Abstract

In the past few decades, great progress has been achieved in the understanding of microbiome-cancer interactions. However, most of the studies have focused on the gut microbiome, ignoring how other microbiomes interact with tumors. Emerging evidence suggests that in many types of cancers, such as lung cancer, pancreatic cancer, and colorectal cancer, the intratumoral microbiome plays a significant role. In addition, accumulating evidence suggests that intratumoral microbes have multiple effects on the biological behavior of tumors, for example, regulating tumor initiation and progression and altering the tumor response to chemotherapy and immunotherapy. However, to fully understand the role of the intratumoral microbiome in cancer, further investigation of the effects and mechanisms is still needed. This review discusses the role of intratumoral bacteria in tumorigenesis and tumor progression, recurrence and metastasis, as well as their effect on cancer prognosis and treatment outcome, and summarizes the relevant mechanisms.

## Introduction

Despite the well-known connection between the microbiome and human health, insufficient attention has been given to the microbiome’s effect on the host, particularly the relationship between tumors and the microbiome (1). All the microorganisms that inhabit the human body, including bacteria, fungi, archaea, viruses and protozoa, form the human commensal microbiota (2). Despite the primary habitat being the gut, thriving microbial populations can be found in areas throughout the body, such as the skin, digestive system, respiratory system, and reproductive system (3). Furthermore, these microbes have the ability to affect a number of significant physiological processes, including metabolism, immunity, and the generation of nutrients (4). There is a complex relationship between microbes and humans that is also present in cancer. The gut microbiota plays a significant role in tumorigenesis and cancer treatment according to a multitude of studies (5–7). Notably, with the improvement of genome sequencing in recent years, researchers have discovered that human solid tumors also have microbiomes, which are called tumor microbiomes (8–10). Although growing evidence shows that there is a strong relationship between intratumoral bacteria and human solid tumors, the specific mechanisms underlying the specific relationship in each cancer remain unclear. This review focuses on the influence of the intratumoral microbiome on tumor occurrence, development, recurrence, metastasis, clinical prognosis and treatment.

It has been suggested that the intratumoral microbiome may be derived from the gut microbiome. By comparing the microbiomes of stool samples, PDAC tumor specimens and nontumor adjacent normal tissues from patients undergoing Whipple surgery, Riquelme et al. found that ~25% of the intratumoral microbiome was derived from the gut microbiome, while there was no trace of the gut microbiome in adjacent normal tissues. This suggests that the gut microbiome is able to specifically colonize pancreatic tumors (11). Subsequently, the researchers transferred microbiotas from patients with advanced PDAC into the intestines of mice by fecal microbial transplantation. Interestingly, they were able to detect human donor bacteria within the tumors of mice after the fecal microbial transplantation, but the bacteria from donors accounted for less than 5% of the bacteria in the intratumoral microbiomes. In addition, they found significant changes in the bacterial composition of the intratumoral microbiomes in mice after the fecal microbial transplantation. These results suggest that the gut microbiome can modulate the intratumoral microbiome, and these changes can be caused in part by direct transfer of gut bacteria but more importantly can be achieved by altering the intratumoral bacterial composition (11).

At a more local level, the commensal microbiota of solid tumor tissues constitutes a significant part of the tumor microenvironment, influencing tumor initiation and progression (5). For instance, Rubinstein et al. demonstrated that colorectal cancer (CRC) cells are stimulated to grow by *Fusobacterium nucleatum*, which can adhere, invade, and initiate oncogenic and inflammatory responses through their distinctive FadA adhesin (12). In addition to its role in tumor initiation and development, the intratumoral microbiome can also promote metastatic colonization in cancer (13). In the study by Fu et al., depletion of intratumoral bacteria significantly slowed lung metastasis of breast cancer without affecting the growth of primary tumors (14). Similarly, a study showed that *Fusobacterium nucleatum* infection may stimulate tumor cells to secrete specific exosomes that can be taken up by healthy cells to promote prometastatic behavior (15). Moreover, the treatment and prognosis of cancer are also affected by the tumor microbiome according to several recent studies (16–18). In CRC, the bacteria in the tumor can alter the biological structure of the chemotherapeutic drug gemcitabine during therapy, thereby enhancing the tumor’s resistance to chemotherapy (19). Using 16S rRNA gene sequencing, Riquelme et al. found that the long-term survival of patients with pancreatic ductal adenocarcinoma (PDAC) increased with the alpha diversity of the tumor microbiota (11). In addition, *Turicibacter* was identified as a potential independent prognostic factor for patients with nasopharyngeal carcinoma, and the relative abundance of this bacteria was negatively associated with progression-free survival in patients with the disease (20). The differences between the effects of commensal microorganisms and those of intratumoral microorganisms on tumor tissues are not always clear and, due to technical limitations, not always easy to distinguish. In this review, we summarize several possible mechanisms by which the intratumoral microbiome affects the biological behavior of tumors and review the role of the intratumoral microbiome in different human solid tumors and the implications for cancer therapy.

## Mechanisms by which the intratumoral microbiome affects tumorigenesis and tumor metastasis

### Intratumoral microbiome and tumorigenesis

**Figure 1:**
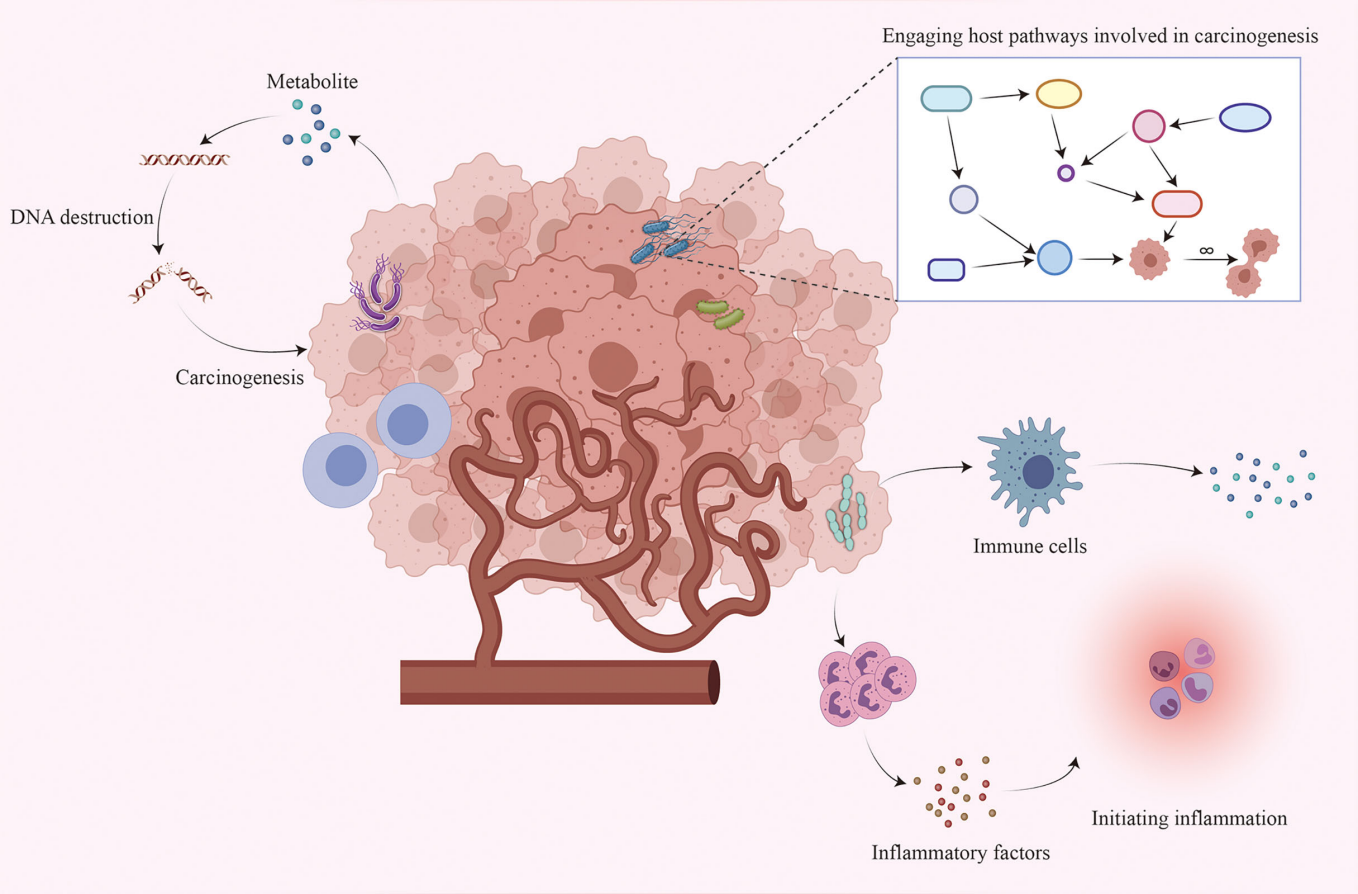
Interaction between the intratumoral microbiome and tumor cells. Although the mechanism by which the intratumoral microbiome promotes tumorigenesis remains unclear, three major mechanisms may contribute to tumorigenesis: increasing the mutation rate by directly destroying DNA, regulating oncogenic signaling pathways, and initiating inflammation and interacting with the host immune system.

Although existing studies have established an inextricable relationship between the intratumoral microbiome and tumorigenesis, the exact mechanisms are not yet fully understood. The following are three possible mechanisms (4, 5, 21, 22): directly promoting tumorigenesis by increasing the mutation rate, regulating oncogenic signaling pathways, and inducing inflammation and altering the local immune microenvironment of the host (**Figure 1**).

Members of the microbiota can produce metabolites such as cytolethal distending toxin (CDT), colibactin and Bacteroides fragilis toxin (BFT) that directly cause DNA damage and trigger mutations (23, 24). Approximately 35% of group B2 *Escherichia coli* isolates possess genomic islands known as polyketide synthetase (pks) islands, which encode colibactin in the form of a biosynthetic nonribosomal peptide synthetase–polyketide synthase hybrid gene cluster (25, 26). Colibactin can lead to double-strand breaks, thereby promoting genome instability and accelerating oncogenesis (23, 27–29). CDT is secreted by some gram-negative bacteria belonging to the ϵ and γ classes of the *Proteobacteria* phylum (30). CDT is a heterogeneous multimeric protein consisting of three subunits (CdtA, CdtB, and CdtC), and CdtB is the primary functional unit causing DNA damage (31–33). Importantly, CdtB acts in a dose-dependent manner, and its effect gradually changes from inducing single-strand DNA breaks to inducing double-strand DNA breaks as the dose increases (34, 35). An abnormal DNA damage responses can lead to genome instability and promote tumor initiation (29). BFT is secreted by *Bacteroides fragilis* and can increase reactive oxygen species and DNA damage by upregulating spermine oxidase to induce colon tumorigenesis (24). Moreover, BFT is capable of inducing the release of PGE2, which induces an inflammatory response by triggering the expression of cyclooxygenase-2, a process intricately linked to the formation of colon cancer (36).

In addition to directly destroying DNA, several microbes possess proteins that affect host pathways, which can cause changes in host signaling and promote tumorigenesis. The Wnt/β-catenin signaling pathway can regulate the biological properties of cells and thus affects cell growth and is altered in many malignancies (37). Significantly, some cancer-related bacteria can affect β-catenin signaling. *Fusobacterium nucleatum* can express the bacterial cell surface adhesion component FadA, which can activate β-catenin signaling by binding to E-cadherin and can differentially modulate immune inflammatory and oncogenic responses, thereby promoting colorectal carcinogenesis (12, 38). The pathogenic product of *Salmonella*, AvrA, is inserted into host cells during infection and affects eukaryotic signaling pathways. It can upregulate β-catenin signaling by decreasing the ubiquitination of β-catenin, increasing the phosphorylation of β-catenin and increasing nuclear β-catenin (39). Enterotoxigenic *B. fragilis* can specifically cleave E calreticulin by secreting BFT, which triggers nuclear β-catenin signaling, thereby enhancing the transcription and translation of the proto-oncogene c-Myc and ultimately promoting the formation of colon tumors (40, 41). In addition to the Wnt/β-catenin signaling pathway, microbes can also contribute to tumorigenesis by affecting the ERK and PI3K signaling pathways. Tsay et al. demonstrated that lung cancer patients can develop enrichment of oral taxa (*Streptococcus* and *Veillonella*) in the lower airways through upregulation of the PI3K and ERK signaling pathways (42).

It has been shown that inflammation is inextricably linked to the development of cancer through a variety of pathways (43). The interaction between the commensal microbiota and the human immune system is in a dynamic balance during healthy periods. This immune system-microbial alliance induces a protective immune response against pathogens when the host organism is functioning optimally. However, once this balance is broken, the microbiota can trigger proinflammatory responses or immunosuppressive programs to influence the body’s immune response to tumors and thus promote tumorigenesis (5, 44). Signal transducer and activator of transcription 3 (STAT3) has been found to promote cell proliferation, differentiation and inhibition of apoptosis (45). For example, the cytotoxin-associated gene A protein-eukaryotic translation elongation factor 1-alpha 1 (CagA−eEF1A1) complex in CagA^+^ *Helicobacter pylori* can affect the activity of STAT3 by recruiting PKCδ, which promotes cell proliferation and tumorigenesis by upregulating the expression of cell cycle regulators and the proto-oncogene MYC (43, 46). In addition, Zhang et al. demonstrated that *Salmonella enterica* serovar *typhimurium* carrying STAT3 siRNA can silence STAT3 to cease cell growth and enhance cell death (47). Of course, apart from STAT3, bacteria can also promote tumorigenesis through other molecules. Jin et al. demonstrated that the lung commensal microbiome can induce the activation and proliferation of Vγ6+Vδ1+γδT cells by stimulating myeloid cells to produce Myd88-dependent IL-23 and IL-1β, IL-17 and other small molecules, which ultimately promote inflammatory responses and neoplastic hyperplasia (10). *Fusobacterium nucleatum* can form an immune inflammatory microenvironment that promotes intestinal tumorigenesis by recruiting tumor-infiltrating immune cells (48). Moreover, the fap2 protein of *Fusobacterium nucleatum* can interact with TIGIT receptors expressed on NK cells and lymphocytes, which can inhibit NK-cell cytotoxicity and T-cell activity, ultimately weakening antitumor immune responses (49). These studies explain the role of bacteria in tumor initiation and provide new insights into potential targets in cancer therapy.

### Intratumoral microbiome and tumor metastasis

**Figure 2:**
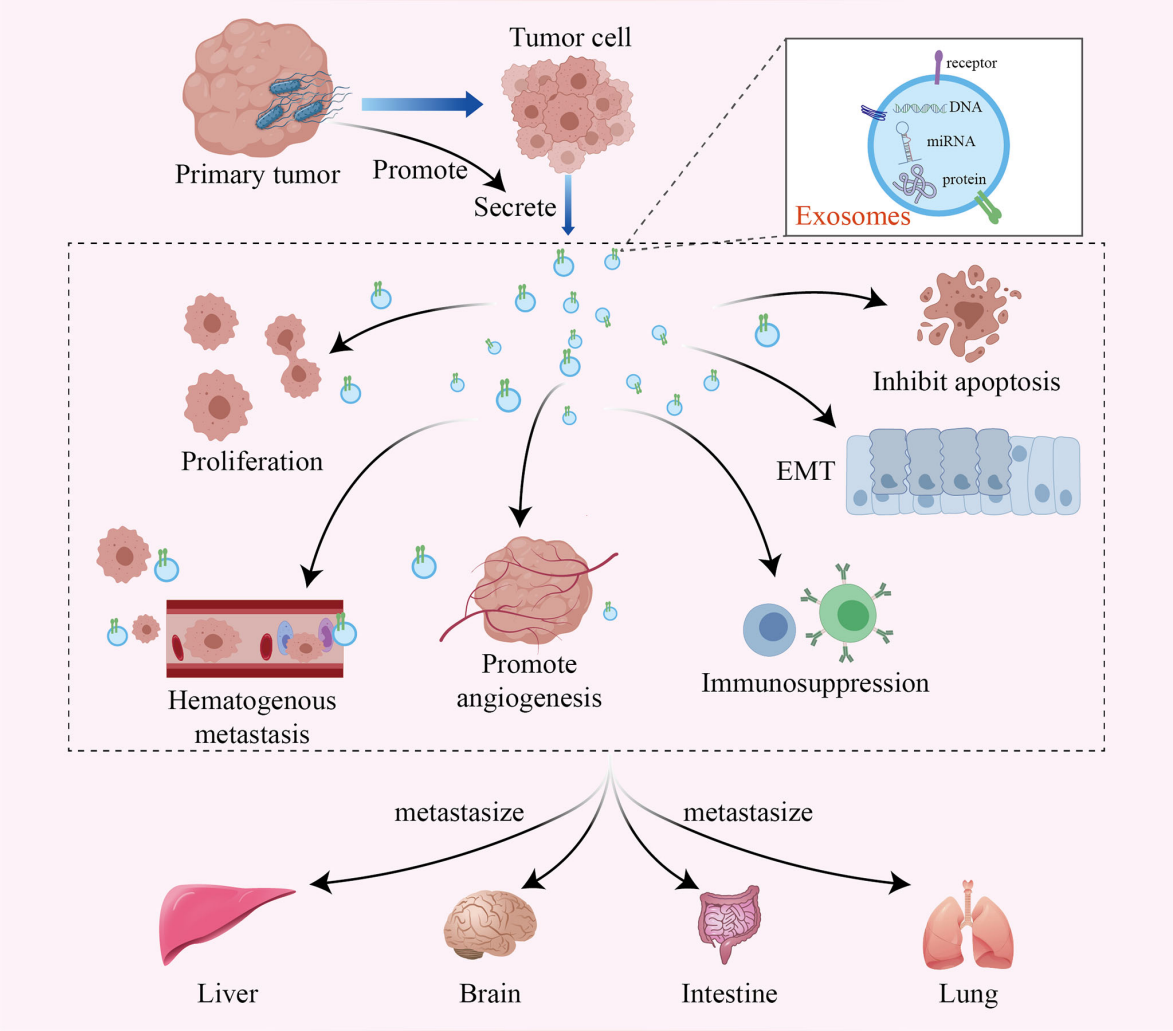
The intratumoral microbiome promotes tumor metastasis by facilitating the production of tumor-derived exosomes. Exosomes are active vesicles carrying various DNAs, miRNAs and proteins. The intratumoral microbiome can cause infected tumor cells to secrete more exosomes, which promote tumor metastasis through various mechanisms, such as remodeling the tumor microenvironment, promoting tumor cell proliferation and inhibiting apoptosis, promoting epithelial-mesenchymal transformation, inhibiting the antitumor immune response, and promoting hematological tumor metastasis and angiogenesis.

Although the specific mechanism by which intratumoral bacteria affect tumor metastasis is still unclear, recent studies have shown that exosomes secreted by infected cancer cells may be one of the mechanisms. Exosomes are 40-100 nm vesicles with 5’-nucleotidase activity that are released by a variety of cultured cells. They bear various proteins, lipids, and RNAs, mediating intercellular communication between different cell types in the body and thus affecting normal and pathological conditions (50–52). Tumor-derived exosomes can transfer miRNAs and proteins to normal tissues and promote tumor metastasis through multiple mechanisms, such as remodeling the tumor microenvironment, promoting tumor cell proliferation and inhibiting apoptosis, promoting epithelial-mesenchymal transformation, inhibiting the antitumor immune response, and promoting hematological tumor metastasis and angiogenesis (53–56) (**Figure 2**). Notably, many studies have shown that tumor cells infected by bacteria may secrete more exosomes, thus accelerating the metastasis of the tumor (15, 57, 58).

*Enterotoxigenic Bacteroides fragilis* (ETBF) can inhibit exosome-packaged miR-149-3p and further promote PHF5A-mediated alternative splicing of KAT2A RNA in CRC cells, which ultimately promotes cell proliferation in CRC (57). The facultative intracellular bacterium *Fusobacterium nucleatum* is an important CRC-associated intratumoral bacterium. It can stimulate tumor cells to generate miR-1246/92b-3p/27a-3p-rich and CXCL16/RhoA/IL-8-bearing exosomes, which can be delivered to uninfected cells to promote prometastatic behaviors (15). Moreover, it has been demonstrated that the activation of Toll-like receptor 4 in tumor cells supports tumor progression by stimulating the release of immunosuppressive exosomes, which allow tumor cells to escape immune surveillance and even play a role in the metastatic process (58). However, our research on the effect of intratumoral bacteria on tumor metastasis is still in its infancy, so we need more research to confirm these findings.

## Intratumoral microbiome and human solid tumors

### Lung cancer

The lung is the largest surface area mucosal organ of the human body, and it is directly connected with the external environment. Therefore, it provides a favorable environment for microorganism colonization. However, lung tissue in healthy people had always been considered sterile in the past. Fortunately, with the emergence of culture-independent 16S rRNA sequencing technologies, a growing number of microbial populations have been found in the lung (59–61). The lung microbiota is quite different from the nasal, oral, skin, gut and vaginal microbiotas. In the healthy lung, *Firmicutes*, *Actinobacteria*, *Bacteroidetes* and *Proteobacteria* are the most abundant phyla, while the core microbiota genera include *Veillonella*, *Haemophilus*, *Neisseria*, *Streptococcus*, *Fusobacterium* and *Prevotella* (60, 62). An increasing number of studies have shown that bacterial dysbiosis in lung tissue is related to lung cancer (60, 63–66).

According to the latest statistical report, lung cancer has the highest mortality rate among human cancers at present, and the number of deaths is 2.5 times that of CRC. It is estimated that approximately 80% of lung cancer cases are caused by smoking (67). Interestingly, among the bacteria found in lung tumors, those with metabolic pathways that degrade chemicals in cigarette smoke are significantly enriched, indicating a close relationship between intratumoral bacteria and tumorigenesis (8). For instance, Lee et al. found that two phyla (TM7 and Firmicutes) were significantly enriched in lung cancer patients. Furthermore, four genera (*Megasphaera*, *Selenomonas*, *Atopobium*, and *Veillonella*) were more abundant in patients with lung cancer (63). How do intratumoral bacteria affect the occurrence and progression of lung cancer?

First, the lung microbiome can directly promote the growth of tumor cells. The regulatory effects of commensal microbes on the occurrence and development of lung cancer are mainly achieved by modification of the local immune environment. Dysregulation of lung microbial communities may promote changes in carcinogenic pathways through specific microbial components (66). The most commonly mutated gene in lung cancer is the cancer suppressor gene TP53 (68), and certain missense mutations in this gene result in enhanced oncogenic ability (69). Greathouse et al. found that patients with squamous cell carcinoma who are smokers have enrichment of Acidovorax. The researchers found that the same genus were further enriched in patients with TP53-mutated squamous cell carcinoma (70). In addition to TP53, the intratumoral microbiome can also promote lung cancer proliferation and invasion by upregulating the phosphoinositide 3-kinase (PI3K) pathway (71). Tsay et al. showed that upregulation of the PI3K and ERK signaling pathways can induce enrichment of oral taxa (*Veillonella* and *Streptococcus*) in the lower respiratory airways of lung cancer patients. These same signaling pathways are also upregulated in airway epithelial cells exposed to Streptococcus, Veillonella and Prevotella *in vitro* (42).

In addition, the lung microbiome can induce local chronic inflammation by promoting the release of chemokines, cytokines, and other proinflammatory factors and ultimately promote cancer progression (5, 62). Because it is directly connected with the external environment due to respiration, the lung is exposed to a multitude of environmental pollutants and airborne microbes, making it a crucial site of interaction between a microbiome and the immune system (62, 72–74). Jin et al. found that when the local bacterial enrichment patterns and microbiota composition changed, myeloid cells could be activated to produce Myd88-dependent IL-23 and IL-1β. These cytokines can induce Vγ6^+^Vδ1^+^ γδ T-cell activation and proliferation, thus producing IL-17 and other cytokines, which ultimately promote the occurrence of an inflammatory response and the proliferation of tumor cells (10). Moreover, after the treatment of mice with aerosolized antibiotics, Le Noci et al. found that the bacterial load in the lungs of mice decreased while the activation of NK cells and T cells increased, which paralleled a significant reduction in the lung metastasis of B16 melanoma. Additionally, the probiotic *Lactobacillus rhamnosus* was found to strongly promote immunity against B16 lung metastases (75). In conclusion, these studies prove that intratumoral bacteria can promote the occurrence and development of lung cancer by inducing inflammation and regulating the local immune response.

### Pancreatic cancer

Pancreatic cancer has a poor prognosis and is one of the most aggressive types of malignancies, with a five-year survival rate of less than 11% despite continuous efforts by researchers and clinicians. The latest data show that pancreatic cancer is the sixth leading cause of cancer-related mortality in China and the third leading cause in the United States (67, 76). Similar to lung tissue, the pancreas was considered sterile in the past, but recent studies have shown that pancreatic tissue also has its own microbiota (77). Pushalkar et al. used 16S rRNA sequencing and found that *Proteobacteria* (45%), *Bacteroidetes* (31%) and *Firmicutes* (22%) were more enriched in pancreatic cancer tissue than in normal pancreatic tissue (78). A growing number of studies have demonstrated that the intratumoral microbiome has a critical impact on the occurrence, progression and prognosis of pancreatic cancer (8, 11, 19, 78–80). This is achieved primarily through the ability of the microbiota to regulate the body’s immune system and metabolize drugs.

Pushalkar et al. found through bacterial ablation techniques that the pancreatic cancer microbiome can promote tumorigenesis by inducing innate and adaptive immune suppression (78). In addition to bacteria, intratumoral fungi can also promote pancreatic tumorigenesis. Aykut et al. found that the number of fungi in human and mouse PDAC was approximately 3000 times higher than that in normal pancreatic tissue. Specifically, *Malassezia* spp. are fungal community members predominantly found in PDAC in humans and mice. The glycans in the cell wall of this fungus can activate the host complement cascade by binding mannose-binding lectin (MBL), thus promoting PDAC. Tumor progression can be prevented when MBL or C3 is absent in tumor cells in the extratumoral region or when c3ar is knocked out (80). Additionally, intratumoral bacteria can also influence the responsiveness of pancreatic cancer to chemotherapeutic drugs. Geller et al. found, through their analysis of human CRC samples, that there was bacterial DNA in 76% of samples, with *Gammaproteobacteria* being the dominant class. The long isoform of the bacterial enzyme cytidine deaminase (CDD_L_) in these bacteria can alter the biological structure of the chemotherapy drug gemcitabine and render it inactive, thus promoting PDAC chemotherapeutic drug resistance (19).

In addition, some intratumoral bacteria also enhance antitumor immunity. For example, by analyzing the intratumoral bacterial composition of PDAC patients with different survival times, Riquelme et al. found that the α-diversity of patients’ intratumoral bacteria increased with increasing survival time and determined an intratumoral microbiome signature (*Bacillus clausii*-*Streptomyces*-*Pseudoxanthomonas*-*Saccharopolyspora*) with the ability to predict long-term survival. The bacteria in this signature facilitate the activation and recruitment of CD8+ T cells, thereby promoting antitumor immune responses (11). Collectively, these findings strongly suggest that the intratumoral microbiome can influence pancreatic cancer progression by modulating the local immune response.

### Colorectal cancer

CRC is the second leading cause of death among cancer patients, with an incidence of 10.2% and mortality of 9.2% (67, 81). Commensal microorganisms in the distal intestine are particularly abundant and diverse (21); the gut bacteria and their byproducts influence the residency and recruitment of myeloid cells in tissues, and these cells promote cancer development by secreting cytokines and attenuating the normal effects of antitumor immunity (82).

An increasing number of studies have confirmed the role of the intratumoral microbiome in CRC. Mechanistically, the bacteria can promote tumorigenesis by directly damaging host DNA, modulating the local immune microenvironment and engaging host pathways involved in carcinogenesis (12, 48, 83). Studies using 16S rRNA gene sequencing, whole-genome sequencing, or transcriptome sequencing have determined that CRC tissue is rich in *Fusobacterium nucleatum* (84–86). Subsequent studies have proven that *Fusobacterium nucleatum* can promote tumor cell proliferation and tumor growth (87). According to Kostic et al., one possible mechanism is that Fusobacterium nucleatum can increase the level of tumor-infiltrating immune cells to create an inflammatory microenvironment conducive to CRC progression (48). In addition, *Fusobacterium nucleatum* can stimulate CRC progression by secreting the FadA adhesin. FadA activates β-catenin signaling by binding E-cadherin and regulates the inflammatory response, ultimately promoting CRC progression (12). Other commensal bacteria, such as ETBF and *Escherichia coli*, have also been shown to be directly associated with colon cancer (88, 89). Wu et al. found that BFT, a toxin secreted by ETBF, can stimulate the transcription and translation of the proto-oncogene c-Myc, thereby triggering persistent cellular proliferation (40). The researchers found that the colibactin secreted by *Escherichia coli* contains a cyclopropane ring, an active structural motif found in natural products inducing DNA alkylation, suggesting that colibactin can alkylate DNA *in vivo* and participate in the development of CRC (90).

Additionally, intratumoral bacteria also have an indispensable influence on the cancer treatment response. For example, Yu et al. found enrichment of *Fusobacterium nucleatum* in tissues of colon cancer patients with recurrence post chemotherapy. In addition, the researchers also found that *Fusobacterium nucleatum* promotes resistance of CRC to chemotherapy. Mechanistically, *Fusobacterium nucleatum* downregulates microRNAs (miR-18a and miR4802) to activate the autophagy pathway and promote chemoresistance in CRC (18). In contrast, another study demonstrated that *Bifidobacterium* accumulates in the tumor microenvironment and promotes immunotherapy efficacy through the STING signaling pathway (91). Overall, tremendous progress has been made thus far in the study of the effects of intratumoral microbes on CRC, but the mechanistic details still need to be explored more deeply to provide potential avenues for the prevention and treatment of CRC.

### Breast cancer

BC is the most frequent malignancy in women worldwide and the most common cause of cancer death (92). Since 2014, the incidence of female BC has been slowly increasing at a rate of 0.5% per year (67). Human mammary tissue is not sterile, and a rich microbiome is also present. Furthermore, the resident microbiomes of healthy breast tissues, BC tissues and even GC tissues of different subtypes are different (93–95). *Lactobacillus, Fusobacterium*, *Hydrogenophaga*, *Atopobium* and *Gluconacetobacter* were enriched in the breast tissue of women with invasive BC compared to normal breast tissue (94). In addition, Banerjee et al. detected *Cardiobacterium*, *Arcanobacterium*, *Escherichia*, *Bifidobacterium* and *Citrobacter* in endocrine receptor-positive BC samples, while *Bordetella*, *Pasteurella, Chlamydia*, *Campylobacter*, *Chlamydophila* and *Legionella* were closely related to triple-positive BC. *Streptococcus* was enriched in human epidermal growth factor receptor 2-positive BC, while *Arcobacter*, *Aerococcus*, *Rothia, Orientia* and *Geobacillus* were related to triple-negative BC (95). These studies reveal the same result: the intratumoral microbiome is inextricably linked to BC. As such, what is its role in BC initiation and progression?

The intratumoral microbiome can promote the growth of BC by activating estrogen signaling, regulating the metabolism of cancer cells, promoting the development of local inflammatory responses and reducing the number of lymphocytes. Furthermore, the intratumoral microbiome can also promote the metastasis and recurrence of BC by supporting cell movement, transforming epithelial cells into mesenchymal cells, and promoting tumor stem cell function (96). Parhi and his colleagues showed that *Fusobacterium nucleatum* colonizes BC *via* Gal-GalNAc, which is abundant in tumor cells, and promotes BC development and metastasis by inhibiting T-cell aggregation in the local tumor tissue (13). Fu et al. studied a mouse spontaneous mammary tumor model and showed that reduction of intratumoral bacteria significantly reduced lung metastasis but did not affect the growth of tumors at the primary site. In addition, the researchers demonstrated that intratumoral bacteria can enhance the resistance of circulating tumor cells to fluid shear stress by reorganizing the actin cytoskeleton, thereby promoting breast cancer metastasis (14).

Several recent studies have shown that the intratumoral microbiome can also have an impact on BC prognosis. For example, by comparing the microbiome composition of different subtypes of BC, Banerjee et al. found that differences in prognosis between different subtypes of BC were strongly associated with a diverse intratumoral bacteria (97). Mechanistically, intratumoral bacteria can influence immune regulatory gene expression, immune cell infiltration, and the release of soluble factors, which may alter the prognostic and clinicopathological features of BC (98). These findings support the idea that the intratumoral microbiome can affect the occurrence and progression as well as prognosis of BC and shed new light on the treatment of BC.

### Genitourinary cancers

Genitourinary tumors include kidney cancer, bladder cancer, prostate cancer, ovarian cancer, and endometrial cancer. Genitourinary tumors are a miscellaneous group of tumors, and the intratumoral microbiome in such tumors has been less studied than those of other malignant tumors. Just as fecal samples are analyzed in the study of CRC, urine must be considered in the study of the microbiomes associated with kidney cancer and uroepithelial carcinoma. Although urine was previously considered sterile, with the development of sequencing technology, data obtained by sequencing methods have initially shown that bacteria are also present in the urine of healthy individuals (99). A few studies have shown that the urine microbiome of patients with bladder cancer differs from that of healthy controls, with the main feature being enrichment of *Fusobacterium*, *Actinomyces* and *Firmicutes* and a decrease in *Streptococcus (* 100, 101). However, whether there is a causal relationship between the microbiomes in bladder tissue and urine and the most common histological type of bladder cancer (uroepithelial carcinoma) still needs to be elucidated. Notably, the relationship between schistosomiasis and squamous carcinoma of the bladder is well established, and preinfection with the pathogen that causes schistosomiasis is a recognized cause of squamous carcinoma of the bladder (102). Renal cell carcinoma tissues have high relative abundances of the *Chloroplast* class and the *Streptophyta* order compared to adjacent normal tissue (103). However, the effect of the microbiome in urine and tissues on the prognosis of uroepithelial carcinoma and renal cancers remains unclear, yet studies have found that intratumoral bacteria in prostate cancer are associated with prognosis.

Ma et al. found that in prostate cancer, intratumoral bacteria such as *Listeria monocytogenes* can directly slow tumor growth by recruiting immune cells and are thus negatively correlated with adverse prognostic features (prostate-specific antigen level, tumor-node-metastasis stage, androgen receptor expression and Gleason score) (104). In addition, some intratumoral microbiomes may influence the therapeutic response of prostate cancer. For example, *Akkermansia muciniphila* can influence the therapeutic response of castrate-resistant prostate cancer patients by regulating abiraterone acetate-mediated microbial community reorganization (105).

It is also important to note that the genital tract microbiome also plays a crucial role in female genital tract malignancies. Ovarian cancer tissue samples have unique bacterial, fungal, viral and parasitic characteristics (106). Similarly, *Atopobium vaginae* and *Porphyromonas* sp. were more highly enriched in endometrial cancer than in healthy tissue (107).

### Other cancers

In addition to these aforementioned major human solid tumors, it is noteworthy that intratumoral bacteria have also been found in other types of cancer, such as esophageal cancer, melanoma, ovarian cancer, bone cancer, liver cancer, and nasopharyngeal carcinoma (8, 20, 108–111). However, the mechanisms by which the intratumoral microbiomes affect these tumors are not well understood.

A recent study showed that *Fusobacterium nucleatum* in esophageal cancer tissue can promote invasion of tumors by activating chemokines such as CCL20 (16). Furthermore, Yamamura et al. found that patients with enrichment of *Fusobacterium nucleatum* in esophageal cancer tissues had shorter survival, suggesting that *Fusobacterium nucleatum* could be used as a prognostic biomarker (16). Similarly, by comparing nasopharyngeal carcinoma patients with different survival times, researchers found that patients with a lower relative abundance of *Turicibacter* had longer progression-free survival (20). In the liver cancer microenvironment, the number of *Pseudomonadaceae* species with antitumor effects is significantly decreased and linearly correlated with the prognosis of patients with primary liver cancer (109). In a study of the microbiotas of normal versus melanoma pig skin, *Trueperella* and *Fusobacterium* genera were found to be enriched in the melanoma samples (112). Nakatsuji et al. discovered through cell culture that a skin commensal microbe (*Staphylococcus epidermidis*) can produce 6-N-hydroxyaminopurine, a DNA polymerase inhibitor that blocks the proliferation of tumor cells, and thus protect against skin cancer (113).

In summary, intratumoral microbiomes are receiving increasing attention for their key roles in regulating tumor progression and influencing cancer prognosis (**Table 1**). In the future, more in-depth investigation of the mechanisms by which intratumoral microbiomes influence the biological behaviors of tumors will improve the precision of cancer diagnosis and aid the development of more effective cancer therapies.

**Table 1:** Microbiotas in different cancer tissues and their effects on corresponding cancer tissues and mechanisms of action.

Tumor type	Associated intratumoral microbiome organism(s)	Proposed mechanism
Lung cancer	*Streptococcus* and *Veillonella* (42, 63, 64, 114)	Upregulates patient ERK and PI3K signaling pathways to promote lung cancer cell proliferation and tissue invasion (42)
	*Prevotella* and *Rothia* (42)	N/A
	*Acidovorax* (70)	Induces tumorigenesis *via* mutations in the tumor suppressor TP53 (70)
	*Thermus* and *Legionella* (60)	N/A
	*Acinetobacter* (64, 115)	N/A
	*Granulicatella adiacens* (64)	N/A
	*Brevundimonas*, *Propionibacterium*, and *Enterobacter* (115)	N/A
	*Megasphaera* (63)	N/A
	*Capnocytophaga* (65)	N/A
Pancreatic cancer	*Malassezia* spp (80).	Activates the host complement cascade by binding MBL, thereby promoting PDAC (80)
	*Gammaproteobacteria* (19)	Induces PDAC chemoresistance by converting the chemotherapeutic agent gemcitabine into its inactive form (19)
	*Pseudoxanthomonas*, *Streptomyces-Saccharopolyspora* and *Bacillus clausii* (11)	Promotes antitumor immune response by recruiting and activating CD8+ T cells (11)
Colorectal cancer (CRC)	*Fusobacterium nucleatum* (12, 18, 48, 84, 85, 87, 116)	Recruits tumor-infiltrating immune cells to create a proinflammatory microenvironment conducive to CRC progression (48)
		Promotes CRC by participating in the regulation of E-cadherin/β-catenin signaling (12)
	*Enterotoxigenic Bacteroides fragilis* (ETBF) (40, 89)	Stimulates the transcription and translation of the proto-oncogene c-myc, thereby triggering persistent cellular proliferation (40)
		Promotes IL-17-mediated inflammation by enriching other bacteria and immune cells in local tumor tissue (89)
	*Escherichia coli* (83, 89, 90)	Promotes CRC by directly damaging the DNA of colonic epithelial cells (90)
Breast cancer (BC)	*Fusobacterium nucleatum* (13)	Accelerates BC progression and metastasis by inhibiting T-cell aggregation in the tumor microenvironment (13)
Esophageal cancer	*Fusobacterium nucleatum* (16)	Promotes the invasion of tumors by activating chemokines such as CCL20 (16)
Nasopharyngeal carcinoma	*Turicibacter* (20)	N/A

N/A, Not applicable.

## Intratumoral microbiome and tumor treatment

**Figure 3:**
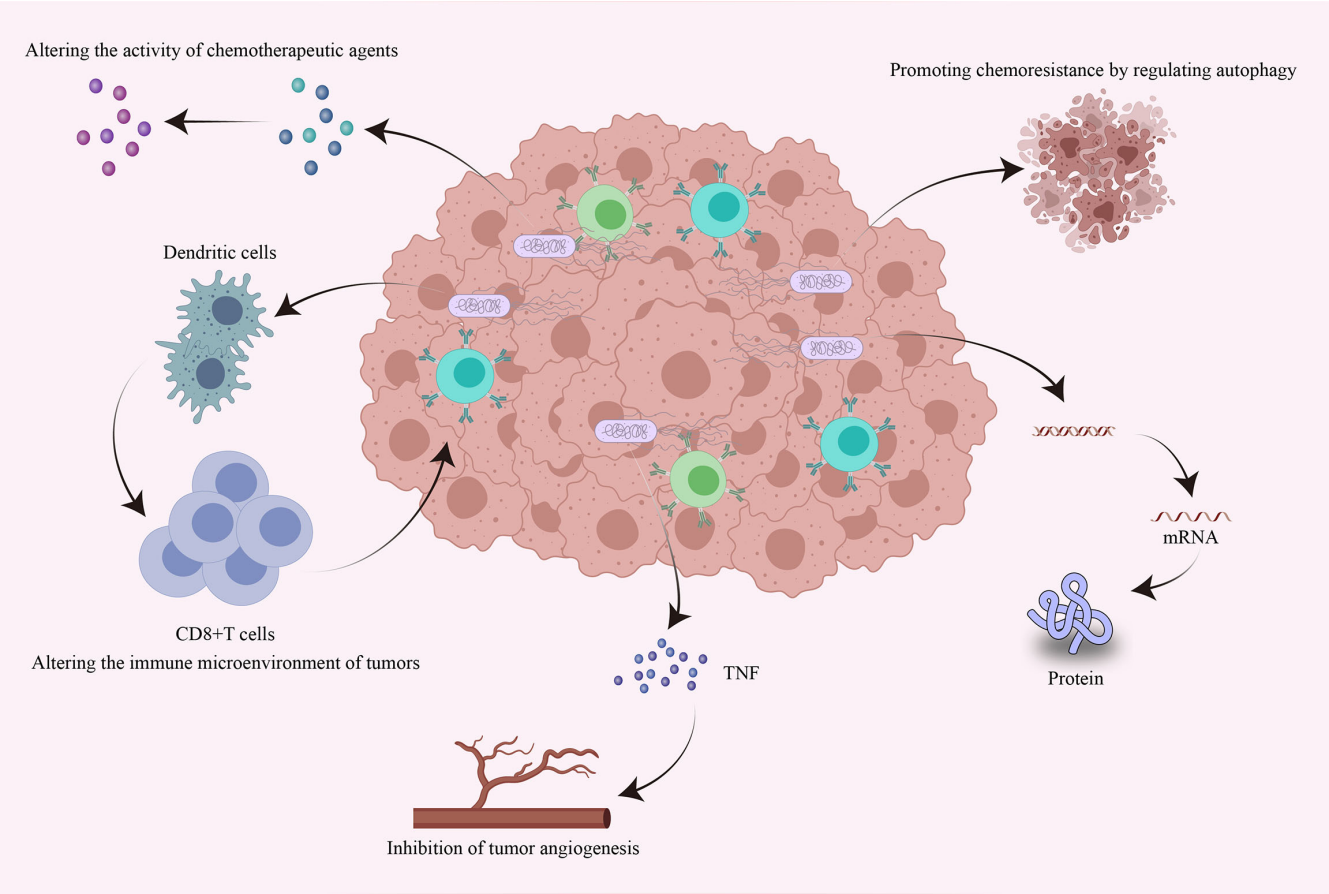
Effects of intratumoral bacteria on tumor chemotherapy and immunotherapy response. Intratumoral bacteria can directly or indirectly influence the efficacy of chemotherapy and immunotherapy through multiple mechanisms. Bacteria accumulating within tumors can directly alter the bioactivity of chemotherapeutic drugs and can promote chemoresistance by regulating autophagy. They can also regulate the expression of specific genes by affecting host signaling pathways. Intratumoral bacteria can promote the production of tumor necrosis factor and thus inhibit tumor angiogenesis. Furthermore, intratumoral bacteria can promote the accumulation of immune cells, thereby enhancing antitumor immune responses and the efficacy of immunotherapy.

At present, the main methods to treat tumors are chemotherapy and immunotherapy. Chemotherapy is administered in the form of genotoxic agents that destroy the DNA of existing tumor cells and prevent new DNA from being generated during proliferation (117). Immunotherapy is mainly achieved by immune checkpoint blockade. Programmed cell death ligand 1 (PD-L1) can reduce the proliferation of T cells by binding to programmed cell death protein 1 (PD-1) on the surface of T cells, thereby inhibiting the body’s antitumor immune response (118). Monoclonal antibodies in immunotherapy can reactivate CD8+ T cells by blocking the PD-1 immune checkpoint on the surface of T cells to induce antitumor responses (119). However, neither of these strategies can completely inhibit tumor growth. Cancer cells can repair DNA damaged by chemotherapeutic drugs, leading to resistance to anticancer therapies (120), while some tumors are not sensitive to immunotherapy. Notably, it has been shown that the intratumoral microbiome can influence the efficacy of chemotherapy by altering the structure of chemotherapeutic agents. In addition, the intratumoral microbiome can also influence the efficacy of immunotherapy by altering the immune environment of the local tumor tissue (121, 122). Next, we mainly discuss the effects of the intratumoral microbiome on chemotherapy and immunotherapy response and the underlying mechanisms (**Figure 3**).

### Effects of intratumoral bacteria on the chemotherapy response

(1) Endogenous enzymes of intratumoral bacteria modify the activity of chemotherapeutic drugs by biotransformation (122). For example, CDD_L_ from *Gammaproteobacteria* can change the structure of the chemotherapy drug gemcitabine, thus causing it to lose its biological activity. Adding the antibiotic ciprofloxacin to the therapeutic regimen can eliminate this effect (19). Similarly, Nemunaitis et al. demonstrated that genetically attenuated *Salmonella* expressing cytosine deaminase can metabolize the antifungal agent 5-fluorocytosine into 5-fluorouracil to treat cancer. Importantly, this treatment is more effective than the chemotherapy drug 5-fluorouracil alone (123) (2). Bacteria can promote chemoresistance by regulating autophagy. *Fusobacterium nucleatum* induces LC3-II expression, autophagosome synthesis and autophagosomal flux in CRC. Thus, *Fusobacterium nucleatum* can stimulate the expression of the autophagy-related proteins ATG7, ULK1 and pULK1 in CRC to promote CRC chemoresistance (18) (3). Bacteria can regulate the expression of specific genes by affecting host signaling pathways. The apoptosis protein inhibitor BIRC3 can inhibit the caspase cascade reaction to reduce apoptosis, leading to chemoresistance in malignancies. *Fusobacterium nucleatum* can activate the Toll-like receptor 4/NF-κB pathway and upregulate the expression of many target genes, such as BIRC3, in CRC cells, thereby promoting the resistance of CRC to 5-fluorouracil (124).

### Effects of intratumoral bacteria on immunotherapy

In addition to chemotherapy, immunotherapy has showed promising effects in recent years and provided new ideas for the clinical treatment of cancer. Anti-PD-1 monoclonal antibodies as well as other immune checkpoint inhibitors have been shown to have promising efficacy (125). Despite this unprecedented efficacy, many patients do not respond, and more worryingly, some patients who show encouraging responses to immunotherapy also develop resistance over time (126). Notably, there is increasing evidence suggesting that intratumoral bacteria can influence the efficacy of immunotherapy (62, 91, 120, 127). Nejman and his colleague showed that the abundance of Clostridium in the melanomas of responders to immune checkpoint inhibition was increased compared with that in patients who did not respond. In contrast, *Gardnerella vaginalis* was more abundant in nonresponder tumors (8). A recent study indicated that commensal *Bifidobacteria* can prime CD8+ T cells and accumulate in the tumor microenvironment by enhancing dendritic cell function, thereby promoting antitumor immunity and anti-PD-L1 efficacy (128). In addition, *Bifidobacteria* can accumulate in the tumor microenvironment, which may facilitate a response to local anti-CD47 immunotherapy in tumor tissues. Mechanistically, *Bifidobacteria* primarily increase dendritic cell crosstalk through stimulation of interferon genes and in an interferon-dependent fashion, ultimately facilitating CD47-based immunotherapy (91). CTLA-4 is a major negative regulator of T-cell activation and exerts inhibitory effects on tumor immunity. Ipilimumab is a monoclonal antibody targeting CTLA-4, and studies have found that Bacteroides fragilis can boost the effects of ipilimumab by promoting Th1 immune responses, which in turn promotes the efficacy of immunotherapy with CTLA-4 blockade (129). Despite numerous studies demonstrating the association of intratumoral bacteria with chemotherapy and immunotherapy efficacy, the mechanisms are still incompletely understood. Therefore, a reassessment of the relationship between chemotherapy and immunotherapy efficacy and intratumoral bacteria will improve the efficacy of cancer treatment. In addition, the combination of genetic engineering and traditional therapies may improve antitumor efficacy and provide new ideas for cancer treatment.

## Conclusion and perspectives

Intratumoral bacteria, as an important component of tumor microecology, are receiving increasing attention. The intratumoral microbiome in solid tumors at different sites in humans plays a similar role to the local tumor microenvironment. Both factors can influence tumor initiation, progression, and response to therapy. In general, the intratumoral microbiome can promote tumorigenesis by directly causing mutations or engaging host signaling pathways. In addition, the intratumoral microbiome can cause inflammation and alter the local immune environment of tumors, thereby promoting tumor cell growth (4, 5, 21, 22, 43).

Notably, it has been shown that intratumoral bacteria can affect the tumor response to therapy through various mechanisms, which provides new insights into the treatment of tumors (18, 91, 119–122, 124). Although we have gained an increasing understanding of the role of the intratumoral microbiome in cancer initiation, progression, and treatment, the intricate relationships between intratumoral bacteria, the tumor, and the tumor microenvironment still require further study. Revealing these relationships may provide more valuable insights into cancer prevention, diagnosis and treatment.

Over the past decades, chemotherapy and immunotherapy have been mainstays of cancer treatment. However, tumors gradually develop resistance to chemotherapeutic drugs, and some patients with encouraging responses to immunotherapy also gradually develop resistance over time. Fortunately, with the development of gene editing technology, new methods for tumor treatment are possible, including genetic engineering. Compared with traditional therapeutic methods, genetic engineering has obvious advantages as tumor therapy. Genetically engineered bacteria are targeted in such a way that they can uniquely target tumors and accumulate in the tumor microenvironment. For example, *Clostridium* (obligate anaerobes) cannot survive in oxygen, and those that enter the body can only colonize anoxic areas (130). Completely deoxygenated tissues do not exist in the vast majority of organs in the body and are unique to tumors. Therefore, obligate anaerobes can very effectively target the tumor area and accumulate to exert their effects (131). Moreover, the effectiveness of genetically engineered bacteria is not affected by genetics, and bacteria can directly access the deep layers of the tumor and thereby kill cancer cells (132).

Furthermore, the combination of genetically engineered bacteria with chemotherapy significantly improves treatment efficacy and reduces toxicity compared to chemotherapy alone (133). Chen et al. found that the combination of triptolide and *Salmonella* VNP20009 significantly increased treatment efficacy in mouse melanoma. Triptolide reduced neutrophil infiltration in melanoma by inhibiting intratumor angiogenesis, which increased the accumulation of *Salmonella* VNP20009 and ultimately created a more hypoxic tumor microenvironment (134). Genetically engineered bacteria can also be combined with immunotherapy to improve efficacy. The bacteria can modify the local immune microenvironment of the tumor by modulating innate and adaptive immune responses, ultimately enhancing the host’s antitumor immune response (135). Despite the promising performance of genetically engineered bacteria in the treatment of cancer, many questions remain. For example, how can bacterial virulence be minimized so that safety is improved? How can the ability of bacteria to accumulate in tumor tissue be enhanced? How can the genetic instability of genetically engineered bacteria be addressed? With the advancement of medical and synthetic biology research, the above problems will be solved, and genetically engineered bacteria will be a novel approach for cancer treatment in the future.

With increasing in-depth study of the tumor microenvironment, evidence indicates that there are bacteria hidden within the tumor and that these intratumoral bacteria are unique and impact tumor initiation, progression, recurrence, metastasis, and prognosis. Moreover, intratumoral bacteria have targeting capabilities and good adaptability, properties that are improve their promise as a new strategy for treating tumors. However, there are still many issues that urgently need to be addressed. For example, do the intratumoral microbiomes of different types of cancer operate *via* different mechanisms of action? Does the intratumoral microbiome originate from the primary site of the tumor or is it derived from other parts of the body? Does tumor progression further promote the accumulation of intratumoral microbes? Overall, microbiome research in oncology is an emerging area worth exploring. Multidisciplinary study of intratumoral bacteria is essential. These challenges will be overcome, and a new era of tumor diagnosis and treatment will emerge.

## Author contributions

ZR and GC designed and reviewed the study. FG, BY, and BR wrote the manuscript. YS, JY, and DW revised the manuscript. All authors contributed to the article and approved the submitted version.

## Funding

This study was sponsored by grants from the National Key Research and Development Program of China (2018YFC2000500 and 2022YFC2303103), Research Project of Jinan Microecological Biomedicine Shandong Laboratory (JNL-2022015B and JNL-2022001A), and National Natural Science Foundation of China (U2004121).

## Conflict of interest

The authors declare that the research was conducted in the absence of any commercial or financial relationships that could be construed as a potential conflict of interest.

## Publisher’s note

All claims expressed in this article are solely those of the authors and do not necessarily represent those of their affiliated organizations, or those of the publisher, the editors and the reviewers. Any product that may be evaluated in this article, or claim that may be made by its manufacturer, is not guaranteed or endorsed by the publisher.

## Abbreviations

CRC, colorectal cancer; PDAC, pancreatic ductal adenocarcinoma; BC, breast cancer CDT, cytolethal distending toxin; BFT, *Bacteroides fragilis* toxin; STAT3, signal transducer and activator of transcription 3; ETBF, enterotoxigenic *Bacteroides fragilis*; MBL, mannose-binding lectin; CDD_L_, long isoform of the bacterial enzyme cytidine deaminase; PD-L1, programmed cell death ligand 1; PD-1, programmed cell death protein 1.
